# Molecular profile of urine extracellular vesicles from normo-functional kidneys reveal minimal differences between living and deceased donors

**DOI:** 10.1186/s12882-018-0985-3

**Published:** 2018-07-31

**Authors:** S. Inés Lozano-Ramos, Ioana Bancu, Laura Carreras-Planella, Marta Monguió-Tortajada, Laura Cañas, Javier Juega, Josep Bonet, M. Pilar Armengol, Ricardo Lauzurica, Francesc E. Borràs

**Affiliations:** 1 REMAR-IVECAT Group, Health Science Research Institute Germans Trias i Pujol, Can Ruti Campus, Ctra. de Canyet s/n, Edifici “Escoles”, 08916 Badalona, Barcelona Spain; 2 Department of Cell Biology, Physiology and Immunology, Universitat Autónoma de Barcelona, 08193 Bellaterra, Cerdanyola del Vallès Spain; 3 Universitat Autónoma de Barcelona, 08193 Bellaterra, Cerdanyola del Vallès Spain; 4 Nephrology Service, Germans Trias i Pujol University Hospital, Carretera de Canyet s/n, 08916 Badalona, Spain; 5 Genomic Platform, Health Science Research Institute Germans Trias i Pujol, Can Ruti Campus, Badalona, Ctra. de Canyet s/n, Edifici “Escoles”, 08916 Badalona, Barcelona Spain

**Keywords:** Extracellular vesicles, Exosomes, Kidney transplantation, Kidney donor, Size-exclusion chromatography

## Abstract

**Background:**

Kidney transplantation (KTx) is the best therapeutic approach for chronic kidney diseases leading to irreversible kidney failure. Considering the origin of the graft, several studies have reported differences between living (LD) and deceased donors (DD) in graft and patient survival. These differences seem to be related to multiple factors including, donor age and time of cold ischemia among others. Many of transplanted organs come from old-aged DDs, in which pre-transplant biopsy is recommended. However, kidney biopsy has several limitations, and there is a need to develop alternatives to assess the status of a kidney before transplantation. As the analysis of urinary extracellular vesicles (uEVs) rendered promising results as non-invasive biomarkers of kidney-related pathologies, this pilot study aimed to investigate whether profiling uEVs of LDs and DDs may be of help to assess the quality of the kidney before nephrectomy.

**Methods:**

uEVs from 5 living donors and 7 deceased donors were isolated by size-exclusion chromatography, and their protein and miRNA content were analysed by liquid chromatography followed by mass spectrometry and next generation sequencing, respectively. Then, hierarchical clustering and venn diagrams were done with Perseus software and InteractiVenn tool. Specific EVs data bases were also used for Gene Ontology analysis.

**Results:**

Next generation sequencing revealed that uEVs from DDs contained less miRNAs than LDs, but most of the DD-expressed miRNAs were shared with LDs (96%). Only miR-326 (targeting the apoptotic-related Bcl2) was found significantly over-represented in LD. Focusing on the protein content, we detected a low intra-group correlation in both types of donors. Despite these differences, hierarchical clustering of either miRNA or protein data could not identify a differential profile between LDs and DDs. Of note, 90% of transplanted patients had a functional graft after a year from KTx.

**Conclusions:**

In this pilot study we found that, in normo-functional grafts, minor differences in uEVs profile could not discriminate between LDs and DDs.

## Background

Chronic kidney disease is a public health issue, causing an important mortality rate and high economic impact [1]. Currently, different replacement therapies for end-stage renal disease include haemodialysis, peritoneal dialysis and kidney transplantation (KTx). Of these, only KTx improves the quality of life of the patients, and is clearly better compared to dialysis regarding patient survival [2–4].

KTx may be performed using organs from living (LD) or deceased donors (DD). Although the number of LDs has increased in the late years [5], the majority of transplanted organs come from aged DDs in a state of neurological death. Some of the most important parameters affecting patient’s survival after transplantation are related to age (of both donor and recipient), previous pathology of the recipient and the extent of the ischemia-reperfusion injury [4, 6–8]. It is widely accepted, and strongly supported, that transplantation from LDs offers better long-term outcomes than deceased organ transplants [9–11]; because of DDs are usually older, have more comorbidities and longer cold ischemia time than LD [10–12]. Moreover, in order to maintain the organs in optimal conditions, hemodynamic stability, adequate oxygenation, the correction of hypothermia, diabetes insipidus and electrolyte disturbances should be corrected by drug administration [13]. For these purposes, DDs might receive dopamine, noradrenalin and vasopressin among other drugs. In addition, specific antibiotic treatment might be administered if infection is suspected [14]. All these maintaining interventions may modify the quality of the organ.

To date, the quality of the organ before nephrectomy has only been estimated by sonographic images and kidney biopsy. In fact, kidney biopsy is recommended in expanded criteria donors, including those aged over 60 years, or showing hypertension, diabetes and in non-beating heart donors [15]. Nevertheless, kidney biopsy requires the invasive sampling of the organ and thus might affect also its quality. Therefore, alternative non-invasive techniques are needed to determine the organ status and predict the KTx outcome.

In this context, Extracellular Vesicles (EVs) have emerged as a source of non-invasive biomarkers for several diseases [16, 17]. In particular, urine EVs (uEVs) are viewed as a subcellular image of the glomerular and tubular systems, and changes in their composition may reflect ongoing events occurring in the renal system [18–20]. In this sense, a growing number of studies have proposed several EV-related biomarkers for kidney dysfunction, graft rejection [18, 21–23], or chronic kidney disease [21, 24, 25]. Also, a recently published study analysed EVs from graft-preservation fluid to predict delayed graft function [26]. However, no reports to date have investigated the uEVs profiles from LD and DD organs to distinguish their quality status prior to transplantation. We aim to profile uEV form living and deceased donors to define their RNA and protein content before KTx.

In this pilot study, we explored the miRNA and protein content of uEVs from LD and DD kidney donors. Using size-exclusion chromatography (SEC) as a minimally altering isolation technique to obtain uEVs [27], we report the great similarity in the molecular profile of uEVs from LDs and DDs. Importantly, this correlated with the kidney function of the transplanted patients, which showed normal renal function 1 year after transplantation. In addition, we have identified several previously non-described uEVs miRNAs.

## Methods

The study protocols were approved by the Clinical Research Ethics Committee of the Germans Trias i Pujol University Hospital and conformed to the principles outlined in the Declaration of Helsinki. No statistical power calculation was conducted prior to the study, size sample was estimated based on previous publications [28–30].

### Urine collection

First morning void urine was collected from living kidney donors (*n* = 5) before undergoing nephrectomy. Urine samples from deceased kidney donors (*n* = 7) were collected directly from the catheter 6 h before surgery was performed. Immediately after collection, urine (70–100 mL) was centrifuged at 600 g for 15 min to eliminate cell and debris and frozen at − 80 °C in the presence of the protease inhibitor AEBSF (0.138 mg/mL; Roche, Basel, Switzerland).

### Urine EV isolation by SEC

Cell-free urine samples were unfrozen overnight at 4 °C and centrifuged at 17,000 *g* for 10 min. The supernatant was kept and the 17,000 *g* pellet was treated with DTT (200 mg/mL, Sigma-Aldrich) for 10 min at 37 °C to release Tamm-Horsfall protein polymers, as previously described [31]. The DTT-treated pellet and the 17,000 g supernatant were mixed and centrifuged again at 17,000 *g* for 10 min. Then, supernatant was concentrated by ultrafiltration, using a 100 kDa cut-off Centricon filter unit (Millipore, Bedford, MA). One mL of the retained volume of concentrated urine was then loaded into a 10-mL sepharose CL-2B (Sigma) SEC column to isolate uEVs. For each sample, 20 fractions of 0.5 ml were collected [32].

### Nanoparticle tracking analysis

Concentration and size distribution of uEVs were determined by Nanoparticle Tracking Analysis (NTA) in a Nanosight LM10–12 (Malvern Instruments Ltd., Malvern, UK) equipped with a 638 nm laser and CCD camera (model F-033). Briefly, samples were diluted 50 to 100 times in PBS to reach optimal concentration for instrument linearity. Three videos of 60s time were recorded for each sample at 24 °C, at a camera level of 16, the camera shutter at 30.02 ms and the camera gain set at 650, as recommended by the manufacturer. Analysis was performed using the NTA software version 3.0.

### Flow cytometry

SEC fractions were incubated with aldehyde/sulfate-latex beads of 4 μm (Invitrogen, Carlsbad, CA). Then, EV-coated beads were labelled with anti-CD9 (Clone VJ1/20), anti-CD63 (Clone TEA 3/18) or polyclonal mouse IgG isotype (Abcam, Cambrige, UK) and as secondary antibody FITC-conjugated goat anti-mouse IgG (Bionova, NS, Canada) was used. Then samples were analysed by flow cytometry (FacsVerse, BD Biosciences San Jose, CA). Singlet beads were gated, and the FITC median fluorescence intensity (MFI) of the EVs-coated beads was calculated for each fraction using the FlowJo software (Tree Star, Ashland, OR) [32, 33].

### RNA analysis

RNA analyses were performed in samples from 5 LDs and 5 DDs. Total RNA was extracted from uEVs using mirCURY kit (Exiqon, Vedbaek, Denmark) following manufacturer’s instructions with the previous published modifications [34]. Then, RNA was precipitated using glycogen (20 mg/mL; Roche); 10% AcNa 3 M, pH 5.2 (Sigma-Aldrich) and 2.5 times (*v*/v) of absolute ethanol. RNA profiling was determined using a high resolution kit (Small RNA kit) in a Bioanalyser 2100 System (Agilent technologies, Santa Clara, CA).

Deep sequencing was performed using TruSeq small RNA, (Illumina, San Diego, CA). An equimolar pool of all samples were run on HiSeq2500 (Illumina) using TruSeq paired end cluster generation (v3 for cBOT).

The DESeq package was used to normalize and analyse differential expression between the miRNAs found [35]. Qualitative analysis was done based on raw counts and a given miRNA was accepted as present when the raw count was of at least 5 copies.

### Proteomic analysis

The uEVs-protein content was analysed in DD (*n* = 5) and LD (*n* = 5) by liquid chromatography following mass spectrometry (LC-MS/MS). Briefly, uEVs proteins were digested (LysC and trypsin) and injected in an Orbitrap XL with a 120 min gradient CID method using a 12 cm column. BSA controls were included both in the digestion and LC-MS/MS analysis for quality control.

The data has been analysed using Proteome Discoverer (v2.0) an internal version of the search algorithm Mascot (www.matrixscience.com) against human database (SwissProt Apr 2015 and UniProt Apr 2015). Peptides have been filtered based on a 5% False Discovery Rate.

### Analysis

Venn diagrams and hierarchical clustering was performed using the InteractiVenn tool (www.interactivenn.net) [36] and Perseus software (1.6.0.2) [37, 38]. Further analyses related to biological function and location were based on specific databases, for EVs: EVpedia [39, 40], Exocarta [41, 42] and Vesiclepedia [43]; for miRNAs: mirbase [44]; and for target prediction: miRDB (mirdb.org/miRDB/) [45].

## Results

### Clinical and epidemiological characteristics of donors

A total of 5 LD and 7 DD were included in this pilot study. The LD group included 2 males and 3 females, with a mean age of 57 years old (ranging from 45 to 69). One of the LDs was receiving treatment for hypothyroidism (Levothyroxine) and dyslipemia (statins). Kidney parameters in LDs revealed normal function and no other pathologies were detected. Urine samples were collected from the first morning urine before kidney extraction. After surgery these donors were further monitored in our hospital for 12 to 26 months, and all of them showed normal kidney function. Mean time of cold ischemia in the LD group was of 2.4 h (ranging from 2 to 4 h).

The DD group included 3 males and 4 females, with a mean age of 67 years old (ranging from 49 to 84). Causes of death were cerebrovascular accident, trauma, pulmonary thromboembolism and cardiac event. Most of DDs received standardized pharmacological treatment to maintain blood pressure and preserve organ function (detailed in Table 1). Four of the DDs showed dyslipemia, while serum creatinine levels of the donors were all between 0.8–3.41 mg/dL (Normal range 0.7–1.3 mg/dL). Urine samples from DDs were obtained 6 hours before the surgery. Kidney biopsy was performed in all of the donors using the Remuzzi scoring system to assess the histopathology [46]. Grafts showed a score between 2 and 5, and all were accepted for transplantation. Mean time of cold ischemia in the DD group was 18.5 h (range 13–22 h).

**Table 1:** Clinical and Epidemiological characteristics of living and deceased donors

	uEV Analysis	Age	Gender	HTA	DM2	DLP	Others	SrCr (mg/dL)	Remuzzi Score		Cause of death	Drugs	ICU Drugs
									RK	LK			
LD1	P,T	40–45	F	–	–	–	–	0.7	–	–	–	–	–
LD2	P,T	55–60	F	–	–	–	–	0.98	–	–	–	–	–
LD3	P,T	65–70	M	–	–	–	–	0.63	–	–	–	–	–
LD4	P,T	50–55	M	–	–	Yes	Hipotirodism	0.93	–	–	–	simvastatin, Levotiroxine	–
LD5	P,T	55–60	F	–	–	–	–	1.02	–	–	–	–	–
DD1	P, T	65–70	F	Yes	–	Yes	–	0.8	3	3	CVA	Olmesartan, Lormetazepam	noradrenaline, furosemide
DD2	P, T	65–70	F	–	Yes	–	obesity	0.8	5	5	Ictus	metformine, insuline	clopidogrelt, amoxiciline-clavulanic
DD3	P,T	80–85	F	Yes	–	Yes	–	1.5	4	5	CVA	bisoprolol,torasemide, lorazepam, sertralin, simvastatin	–
DD4	T	75–80	M	Yes	Yes	Yes	–	1.8	4	4	Trauma	Triflusal;enalapril; Metformin; Gliclazide; Ezetimibe; Allopurinol; Glucosamine; Tenoxicam; Omeprazole	manitol, actocortine, noradrenaline,
DD5	T	55–60	M	Yes	–	–	–	1.56	2	–	Cardiac event	Losartan, Atorvastatin, fenofibrate	propofol, atropine, midazolam, noradrenaline
DD6	P	45–50	M	–	–	–	Bone cyst	3.41	2	2	PTE	oxcarbazepine, diazepam, Celecoxib.	adrenaline
DD7	P	70–75	F	–	–	Yes	osteoporosis	0.73	3	2	Ictus	Calcium	noradrenaline

A summary of the age, gender, diseases, cause of death and medication of all the patients is included in this Table. P: sample used for proteomic assays. T: sample used for NGS assays

*F* female, *M* male; *HTA* Hypertension, *DM2* Diabetes Mellitus type 2, *DLP* Dyslipemia, *CVA* cerebrovascular accident, *PTE* Pulmonary thrombo-embolism, *SrCr* Serum Creatinine, *RK* right kidney, *LK* left kidney

### Transplantation details and graft outcome after transplantation

The study is based on a cohort of 12 donors (5 LD and 7 DD). Two organs from DD were not transplanted due to thromboembolism (1 organ) and intra-operatory decision of the surgeon (1 organ), thus a total of 17 kidney transplants (5LD + 12DD) were performed (summarized in Table 2). Stable kidney function in LD was established at median of 5.5 days (range 5–8 days) while it was achieved at median of 17.5 days (range 6–30 days) in DD recipients. Among all transplanted organs, one patient (graft from DD7) presented delayed graft function and another (graft from LD4) never attained a functional graft due to acute cellular rejection. Also, a patient (graft from DD6) developed acute cellular rejection but attributable to lack of adherence to the immunosuppressive treatment. Additionally, two patients needed graft biopsies which revealed respectively anti-calcineurinic toxicity and interstitial fibrosis and tubular atrophy. Chronic humoral rejection was absent in all patients. All the relevant information of the graft outcome is detailed in Table 3.

**Table 2:** Sample characterization

	Living donor	Deceased Donor
Urine samples	*n* = 5	*n* = 7
Kidneys available for KTx	*n* = 5	*n* = 14
Kidneys transplanted	*n* = 5	*n* = 12
Functional organs (1 year after KTx)	*n* = 4	*n* = 11

Sample size details from urine sample collection to recipients outcome

**Table 3:** Clinical characteristics of kidney receptors

Donor	uEV Analysis	Cold ischemia (hours)	Mismatch			Recipient’s previous KTx	DSA	IS	Delay Graft function	ACR	CHR	KB	Days until stable Renal function	SrCr (mg/dL) 1 year After KTx
			A	B	DR									
LD1	P, T	2	0	0	0	1	–	P, B, FK, MMf	–	–	–	–	5	1
LD2	P, T	2	1	1	1	–	–	P, B, FK, MMf	–	–	–	–	6	1.2
LD3	P, T	2	1	1	2	–	–	P, B, FK, MMf	–	–	–	–	8	1.14
LD4	P, T	2	1	1	1	–	–	P, B, FK, MMf	Yes	Yes	–	ACR	–	–
LD5	P, T	4	1	2	1	–	–	P, B, FK, MMf						
DD1	P, T	20	2	2	1	–	–	P, FK, MMf	–	–	–	–	14	1,82
		Not transplanted												
DD2	P,T	18	2	2	1	–	–	P, B, FK, MMf	–	–	–	–	20	1.77
		22	0	0	1	–	–	P, B, FK, MMf	–	–	–	–	15	1.23
DD3	P,T	18	1	1	1	–	–	P, FK, Th, Everolimus	–	–	–	–	7	1.45
		Not transplanted												
DD4	T	20	1	1	1	–	–	P, B, FK, MMf	–	–	–	CNTI	15	1.67
		15	1	2	1	–	–	P, B, FK, MMf	–	–	–	ATN, TAIF	6	2.6
DD5	T	20	1	2	2	–	–	P, B, FK, MMf	–	–	–	–	9	1.64
		17	1	1	1	–	–	Eculizumab, P, FK, MMF, Th	–	–	–	–	24	1.39
DD6	P	13	2	2	1	–	–	P, B, FK, MMf	–	Yes	–	ACR	24	1.03
		22	1	1	1	–	Yes	P,FK; MMf, Th	–	–	–	–	25	1.16
DD7	P	17	2	2	1	–	–	P, B, FK, MMf	Yes	–	–	–	30	1.01
		20	1	1	1	–	–	P, B, FK, MMf	–	–	–	–	30	1.01

A summary of the HLA-mismatch, donor specific antibodies (DSA), cold ischemia time, previous transplants, immunosuppression treatment, delay graft function and renal biopsy are provided. P: sample used for proteomic assays. T: sample used for transcriptomic NGS assays

*KTx* Kidney transplantation, *DSA* donor specific antibodies, *IS* immunosuppression treatment, *ACR* acute cellular rejection, *CHR* chronic humoral rejection, *KB* kidney biopsy, *P* Prednisone, *FK* Tacrolimus, *MMf* mycophenolate mofetil, *B* Basiliximab, *Th* Thymoglobulin, *ATN* Acute tubular necrosis, *CNIT* calcineurin inhibitor toxicity, *TAIF* tubular atrophy and interstitial fibrosis, *AS* arteriosclerosis

One year after transplantation, 4 of 5 grafts from LD and 11 of 12 grafts from DD were functional. One year after KTx, serum creatinine levels were between 1.1–1.2 mg/dL in the recipients of LD kidneys and 1.01–2.6 mg/dL in the recipients of DD kidneys (normal range 0.7–1.3 mg/dL).

### Isolation of uEVs from LDs and DDs

**Fig. 1:**
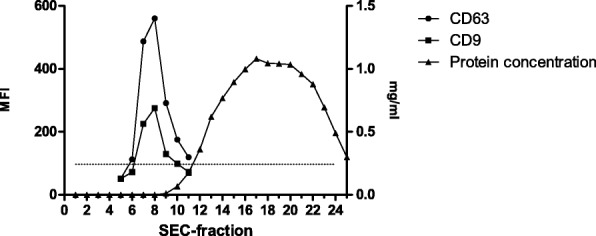
Representative elution profile of uEVs by size-exclusion chromatography (SEC). The expression of CD63 and CD9, depicted as the median fluorescence intensity (MFI, left axis), indicates uEVs presence in SEC fractions. Isotype control is depicted by a dotted line. The total protein content (mg/ml; right axis) was measured in each SEC fraction

SEC-fractions were collected and analysed for tetraspanin markers as a direct evidence of uEV presence. Figure 1 shows the typical SEC elution profile obtained, in which uEVs eluted in fractions 7 to 9 according to the highest fluorescence intensity for CD9 and CD63. uEV samples from both LDs and DDs had the same distribution and no changes were detected either for CD9 or CD63 MFI levels (data not shown). From each sample, three chromatographic fractions containing uEVs (those showing the highest level of CD9 and CD63) were pooled in a final volume of 1.5 mL, and all the subsequent experiments were performed using these pooled fractions.

Regardless of the type of donor, NTA analyses revealed that uEVs had a size distribution modal range from 124 to 250 nm with a mean of 223 ± 30 nm. Particle concentration was well above 10^10^ particles/mL in all samples, showing no differences between both groups. All together these data indicated that uEVs from LDs and DDs did not differ in terms of concentration, size distribution or presence of well-defined EV markers (data not shown).

### RNA content of uEVs

**Fig. 2:**
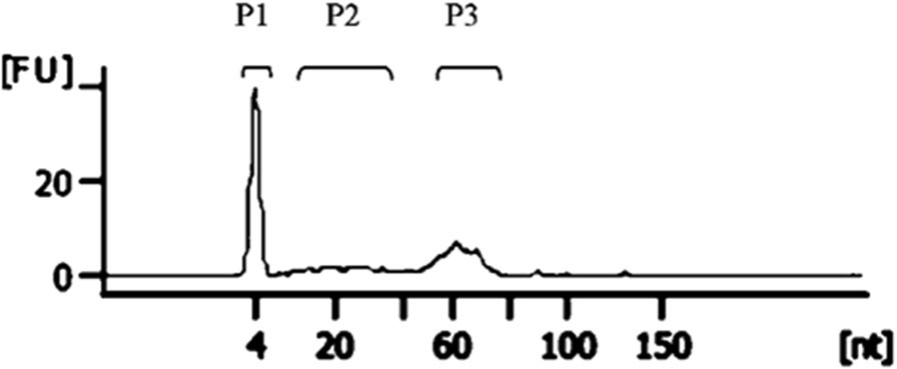
Representative profile of Small RNA from uEVs isolated by size-exclusion chromatography. Total RNA extracted from pooled uEVs profiled by small RNA gel electrophoresis showed a main peak at 60 nucleotides (P3) and a minor peak of RNA around 10–40 nucleotides (P2). The lower marker of the small RNA kit can be observed at 4 nucleotides (P1). A representative experiment is shown

The RNA content of uEVs was analysed in 5 LDs and 5 DDs. RNA species of < 200 nucleotides (nts) were detected in all samples of both groups (Fig. 2). Of note, the RNA profile analysis showed a high variability in the amount of RNAs found in the different samples, independently from the type of donors.

NGS results showed that miRNAs comprised around the 30% of the total RNA content of uEVs, while the remaining 70% was distributed among different RNA species, including tRNA (20%), unmapped RNAs (15%), and non-sense RNA (10%). Also, rRNAs and a minor proportion of protein coding RNAs (< 10%) were found. Given their relevance in cell regulation, miRNAs were further analysed [47, 48].

**Fig. 3:**
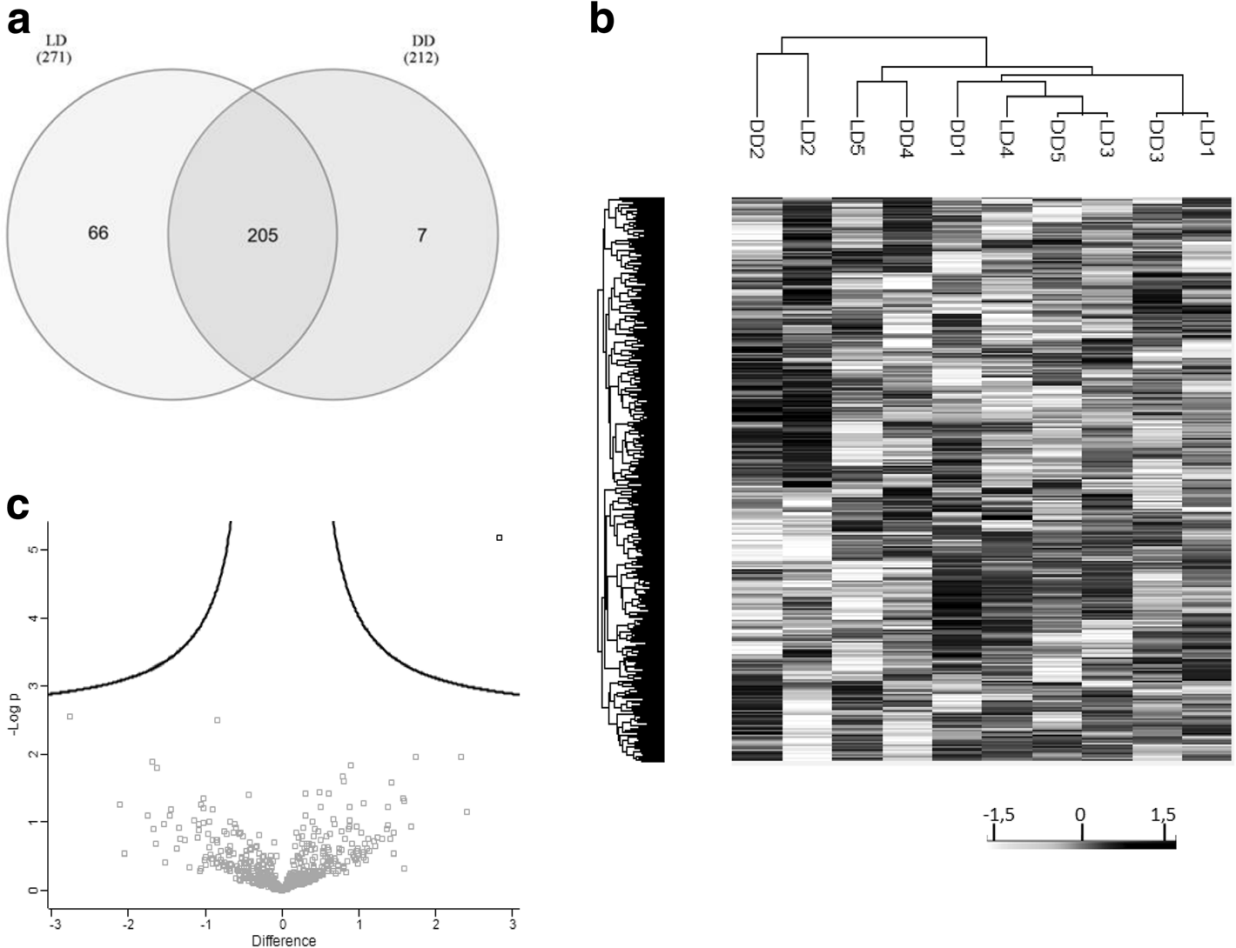
miRNA analysis of uEV from living and deceased donor. **a** Venn diagrams showing the shared and differential miRNA content in uEVs from LD (*n* = 5) and DD (*n* = 5) detected by next generation sequencing. 205 miRNAs overlap amongst the different samples analysed of LD and DD. Only the miRNAs shared within all donors of each group were taken for analysis. **b** Hierarchical clustering analysis of LD and DD uEV samples. Differential analysis of the miRNA content of uEVs from LD and DD. **c** Scatter plot analysis of miRNAs found in uEVs from LD and DD. Dot plot representing the -log of the *p*-value and the difference between the miRNA expression of LD and DD groups. Interestingly, only one miRNA was identified as statistically over-represented in LD (in bold, *p* < 0.05)

A deeper analysis of miRNA content showed a high correlation between samples regardless of the group (R of Pearson 0.96 ± 0.02). The shared and unique miRNAs found in LD and DD groups can be seen in the Venn Diagram shown in Fig. 3a. Up to 205 miRNA sequences were found to be present in all samples, from which a 5.4% (*n* = 10) have not been previously identified in EVs according to specific databases [39, 42, 43] (Table 4). These miRNAs were analysed with a target prediction tool (mirdb.org/miRDB/) [45] and most of them are predicted to target genes related with the urinary tract such as ion channels (SLCs) and aquaporins (AQ), among others.

**Table 4:** miRNAs found by size-exclusion chromatography not described in vesicle-specific databases

miRNA ID	
hsa-miR-7977	
hsa-miR-1260a	
hsa-miR-210-5p	
hsa-miR-653-5p	
hsa-miR-3605-3p	
hsa-miR-203a-3p	
hsa-miR-888-5p	
hsa-miR-152-3p	
hsa-miR-874-3p	
hsa-miR-598-3p	

miRNAs found by size-exclusion chromatography and shared between LDs and DDs (*n* = 205) were compared with those reported in specific databases (EVpedia, Exocarta and Vesiclepedia). Those miRNAs not previously reported in EVs databases are listed

On the other hand, despite the good correlation found, a higher number of miRNAs was found in LDs compared to DDs (a total of 66 sequences, 24% of miRNAs found in LDs). To further investigate the potential role of the miRNAs exclusively detected in LDs (*n* = 66) and DDs (*n* = 7), these miRNAS were analysed by target prediction analysis. A total of 4225 potentially targeted genes were identified for LD miRNAs (target score over 90%), while 367 potentially targeted genes were identified for DD miRNAs. GO analyses of these potential targets revealed that, in comparison to the DD group, the LD group showed an overrepresentation of genes involved in cell adhesion, vesicle mediated transport and cytoskeleton organization, while genes related with regulation of the metabolism were underrepresented.

To investigate whether these differences could segregate different patterns of expression specifically related to the type of donor (which could be of relevance for the graft outcome), hierarchical clustering of miRNAs found was performed (Fig. 3b). Interestingly, no aggregation was detected, meaning that both types of samples showed a similar profile. Moreover, volcano plot revealed only one miRNA differentially overexpressed in LD (miR-326; *p* < 0.05) (Fig. 3c). Despite the differences found in the content of miRNAs, the analyses revealed similar profiles between uEVs from LD and DD.

### Proteomic content of uEVs

Analogously to RNA studies, the proteomic profile was determined on uEVs from LDs and DDs (*n* = 5 each). Unfortunately, two of the samples of the DD could not be used for proteomic analysis due to rather low protein content, and were replaced by two different samples (not previously analysed for miRNA).

Only proteins identified by at least two unique peptides were considered in the analysis and raw data was cleaned up from known contaminants such as keratins and Tamm-Horsfall protein. Under these conditions, more than 500 proteins were identified in both groups.

**Fig. 4:**
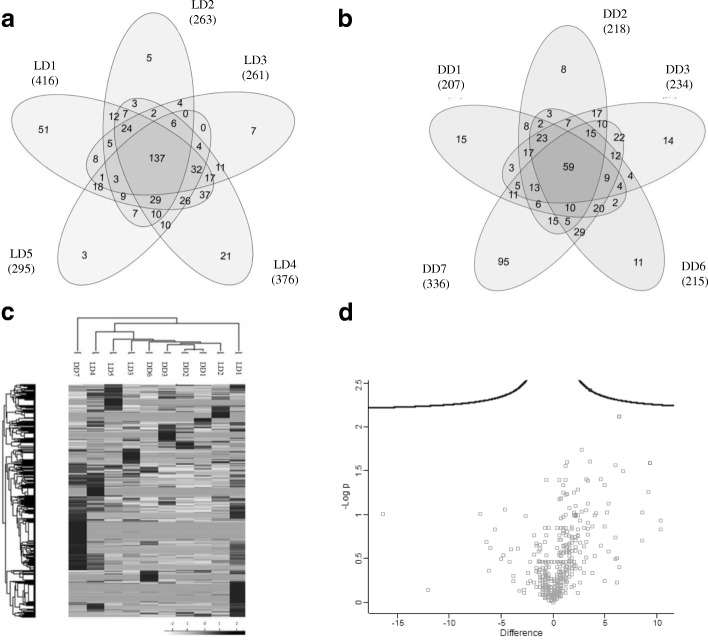
Protein analysis uEV from living and deceased donor by LC/MS-MS. Venn diagrams showing the overlap of proteins detected in uEVs from LD (**a**) and DD (**b**). **c** Hierarchical clustering analysis according to the protein content of uEVs from LD and DD. The analysis did not segregate samples in their corresponding group. **d** Scatter dot plot analysis of the protein content of uEVs from LD and DD. Scatter plot representing the -log of the *p*-value and the differences between LD and DD group

Of note, intra-group Pearson correlation values were lower in the DD group (R values 0.53 ± 0.22) compared with the LD group (0.61 ± 0.12), indicating a higher heterogeneity amongst the DD samples. This was also depicted by Venn diagrams showing up to 137 proteins shared by all LDs (Fig. 4a) while only 59 proteins were shared by all DDs (Fig. 4b). Most of the proteins shared by all DDs (83%, 49 proteins) were also found in all LDs. The majority of these coincident proteins have been previously described as related to EVs, including Ezrin (EZR), Galectin-3-binding protein (LGALS3BP) and Annexins (ANXA). In addition, proteins related to the urinary system such as Neprilysin (NEP), Aminopeptidase N (ANPEP), Aquaporin-1 (AQP1) and several ion transporters are also be found in both groups.

When these results were further compared to investigate whether a different uEVS proteomic profile could not be detected between LD and DD, neither hierarchical clustering nor differential expression analyses revealed a pattern or proteins differentially expressed by LDs and DDs (Fig. 4c and d, respectively). This lack of clear segregation among samples indicates a similar uEVs profile regardless the donor’s origin.

## Discussion

In this pilot study, we aimed to characterise the miRNA and protein profiles of SEC-isolated uEVs from living and deceased kidney donors to profile the organ status before Kidney transplantation (KTx). Several factors account for the outcome of KTx, including those related to the characteristics of the donor [2–4, 11]. Although one of the most important factors directly affecting KTx outcome is the time of cold ischemia, which is usually longer in DD organs, an additional factor to be considered is the status of the organ before the extraction. This could be affected by several factors such as age, donor general condition, or cause of death in DDs. In these aspects, the cohort of patients included in this pilot study shared the same characteristics of larger reported cohorts in terms of mean age and cold ischemia time in DD.

Acceptance of a given organ for transplantation is currently based on clinical and histopathological parameters. The latter are based on a kidney biopsy prior to transplantation, performed only under some circumstances -such as donors aged > 60 years old, or suffering from cardiovascular disorders-, with the aim to verify the quality of the kidney. Yet, as biopsy sampling variability and differences in criteria of analysis may lead to different assessment of the quality of the organ [49, 50], gathering additional information (such that found in uEVs) may be of help defining clearer criteria for organ acceptance.

Given that organ transplant donor characteristics are evolving to older donors (most of them with chronic pathologies) pre-transplant biopsy in these expanded criteria donors is highly recommended to evaluate the state of the organ. As kidney biopsies have limitations, there is a need to improve the analysis of the kidney status prior to transplantation. In this context, the content of uEVs that come from the excretory system may be of interest. Several studies have already shown that urinary EVs may contain specific signatures which may potentially serve as biomarkers of disease [20]. Similarly, normal kidneys also produce EVs, and their specific profile may be indicative of the physiological status of each particular organ. Profiling some specific markers found in urinary EVs may be of clear interest in this particular scenario as a source of non-invasive diagnostic and prognostic biomarkers on the kidney status before nephrectomy.

Most of uEV-related parameters, such as size, concentration and the presence of classical EV markers -including tetraspanins CD63 and CD9- were equally detected in LD and DD samples. Moreover we did not detect in our DD-uEVs preparations any contamination of apoptotic bodies-such as the presence of histones-, which could be expected after the inflammatory state produce by brain death [13, 51]. In this sense, the absence of plasma EVs markers (such as CD5L or moesin), supports that urinary derived EVs are mainly analysed.

Focusing on the study of the RNA content of SEC-isolated uEVs, and in line with previous studies based on ultracentrifuged samples [52], miRNAs and, to a lesser extent, tRNA and rRNA, were among the most abundant RNA species in uEVs. Given the prominent role of miRNAs in cell communication and regulation, we focused on their profile in LD and DD. Although most miRNAs found in DDs were also present in LDs, up to 24% of miRNAs (mainly related to cell communication and signal transduction) were detected only in LDs. The miRNA target prediction showed that those genes related with cell adhesion, vesicle mediated transport and cytoskeleton organization were overrepresented in LD compared to DD, while genes related to regulation of metabolism were underrepresented. These data may be suggestive of an alteration in intracellular trafficking and metabolism in DD. Yet, due to the complexity of miRNA regulation processes and the multiples targets described for a given specific miRNA, further studies are needed to reveal the functional implication of each of them in the specific nephrologic context.

In fact, only one miRNA (miR-326) was found to be over-represented in LD group. miR-326 was described previously in EVs derived from cancer cell lines [53], plasma [54] and endothelial cells [55], and it has been described to target bcl–xL, a member of the bcl-2 family, inducing apoptosis in human platelets [56]. It has also been reported that down-regulation of miR-326 may be involved in chemoresistance in lung cancer [57], poor prognosis and metastatic gastric cancer [58, 59] and osteosarcoma [60]. But the presence of this miRNA was not found before in uEVs and its role in the urinary track remains unknown.

Additionally, we further detected eleven miRNAs not described previously in EVs databases which are predicted to target genes related with the urinary tract. Once again, SEC is confirmed as confident method for isolating uEVs isolated and can be used for EV-related RNA studies and points out to their relevance in normal kidney function.

Focusing on the proteomic studies, and as seen in the miRNA data, a first observation was the identification of higher number of proteins in LDs compared to DDs. In addition, intra-group analyses demonstrated a higher level of variability of protein expression among DDs, which could be related to intrinsic or extrinsic factors such as the donor-conditioning regime. Despite the minimal differences detected between LD and DD in the proteomic and miRNA analysis, in this study all transplanted organs were functional 1 year after KTx, indicating that these minimal changes seem not to be of major relevance for organ function.

Currently, the majority of transplanted kidneys are obtained from aged DDs in a state of neurological death. The use of drugs and medical intervention to maintain these donors in optimal conditions may alter the organ, as suggested by Nemati et al. [61]. Our results show that, before nephrectomy, uEVs derived from DDs showing a 1 year follow-up normal functioning kidneys, contained minimal alterations in their miRNA composition compared to uEVs from LDs. On the other hand, although the DD protein content was highly heterogeneous; such differences did not correlate with the short-term (1 year) graft survival. Additional studies, in a larger cohort of donors and recipients, are needed to investigate whether the information contained in uEVs from subclinical altered kidneys could anticipate any detrimental effect on the graft after transplantation, as recently suggested using preservation fluids [26]. Despite there are some limitations in our pilot study, such as a reduced number of samples and a high variability found among donors, we have successfully profiled uEVs from kidney donors. These results although preliminary, open the possibility to analyse donor uEVs in search of potential biomarkers of kidney quality before nephrectomy.

## Conclusions

Based on uEVs miRNA and proteomic data, no major differences could be detected between LDs and DDs, suggesting that the physiological status of the well-functioning organs before nephrectomy was similar in both groups.

## Abbreviations

BSA: Bovine serum albumin
DD: Deceased donor
EVs: Extracellular vesicles
KTx: Kidney transplantation
LD: Living donor
miRNA: Micro ribonucleid acid
NGS: Next generation sequencing
NTA: Nanoparticle tracking analysis
SEC: Size exclusion chromatography
uEVs: Urine extracellular vesicles

## References

[1] Matsushita K, van der Velde M, Astor BC, Woodward M, Levey AS, de Jong PE (2010). Association of estimated glomerular filtration rate and albuminuria with all-cause and cardiovascular mortality in general population cohorts: a collaborative meta-analysis. Lancet Elsevier Ltd.

[2] Schnuelle P, Lorenz D, Trede M, Van Der Woude FJ (1998). Impact of renal cadaveric transplantation on survival in end-stage renal failure: evidence for reduced mortality risk compared with hemodialysis during long-term follow-up. J Am Soc Nephrol.

[3] Port FK, Wolfe RA, Mauger EA, Berling DP, Jiang K (1993). Comparison of survival probabilities for dialysis patients vs cadaveric renal transplant recipients. JAMA.

[4] Ojo AO, Port FK, Wolfe RA, Mauger EA, Williams L, Berling DP (1994). Comparative mortality risks of chronic dialysis and cadaveric transplantation in black end-stage renal disease patients. Am J Kidney Dis [Internet].

[5] SRTR -- Scientific Registry of Transplant Recipients. Available from: http://www.srtr.org/

[6] Nogueira JM, Haririan A, Jacobs SC, Cooper M, Weir MR (2010). Cigarette smoking, kidney function, and mortality after live donor kidney transplant. Am J Kidney Dis.

[7] Gjertson DW, Cecka JM (2000). Living unrelated donor kidney transplantation. Kidney Int.

[8] Sapir-Pichhadze R, Young A, Joseph KS (2013). Living donor age and kidney transplant outcomes: an assessment of risk across the age continuum. Transpl Int.

[9] Chkhotua AB, Klein T, Shabtai E, Yussim A, Bar-Nathan N, Shaharabani E (2003). Kidney transplantation from living-unrelated donors: comparison of outcome with living-related and cadaveric transplants under current immunosuppressive protocols. Urology.

[10] Hariharan S, Mcbride MA, Cherikh WS, Tolleris CB, Bresnahan BA, Johnson CP (2002). Post-transplant renal function in the first year predicts long-term kidney transplant survival. Kidney Int.

[11] Guimarães J, Araújo AMM, Santos F, Nunes CSS, Casal M (2015). Living-donor and Deceased-donor Renal Transplantation: Differences in Early Outcome–A Single-center Experience. Transpl Proc Elsevier Inc.

[12] Cecka JM. The UNOS renal transplant registry. Clin Transpl. 2001;1–18. 12211771

[13] Van Der Hoeven JABB, Molema G, Ter Horst GJ, Freund RL, Wiersema J, Van Schilfgaarde R (2003). Relationship between duration of brain death and hemodynamic (in)stability on progressive dysfunction and increased immunologic activation of donor kidneys. Kidney Int.

[14] Darby JM, Stein K, Grenvik A, Stuart SA (1989). Approach to management of the heartbeating “brain dead” organ donor. JAMA.

[15] Randhawa P (2001). Role of donor kidney biopsies in renal transplantation. Transplantation.

[16] Azmi AS, Bao B, Sarkar FH (2013). Exosomes in cancer development, metastasis, and drug resistance: a comprehensive review. Cancer Metastasis Rev.

[17] Salih M, Zietse R, Hoorn EJ (2014). Urinary extracellular vesicles and the kidney: biomarkers and beyond. Am J Physiol Renal Physiol.

[18] Peake PW, Pianta TJ, Succar L, Fernando M, Pugh DJ, McNamara K (2014). A comparison of the ability of levels of urinary biomarker proteins and exosomal mRNA to predict outcomes after renal transplantation. Ashton N, editor. PLoS One.

[19] Zhang W, Zhou X, Zhang H, Yao Q, Liu Y, Dong Z (2016). Extracellular Vesicles in Diagnosis and Therapy of Kidney Diseases. Am J Physiol Ren Physiol.

[20] Gamez-Valero A, Lozano-Ramos SI, Bancu I, Lauzurica-Valdemoros R, Borrãs FE. Urinary Extracellular Vesicles as Source of Biomarkers in Kidney Diseases. Front Immunol 2015;6:6. 10.3389/fimmu.2015.00006 PMC4311634 25688242

[21] Zhang X, Nagaraja HN, Nadasdy T, Song H, McKinley A, Prosek J (2012). A composite urine biomarker reflects interstitial inflammation in lupus nephritis kidney biopsies. Kidney Int.

[22] Alvarez S, Suazo C, Boltansky A, Ursu M, Carvajal D, Innocenti G (2013). Urinary exosomes as a source of kidney dysfunction biomarker in renal transplantation. Transplant Proc.

[23] Dimuccio V, Ranghino A, Praticò Barbato L, Fop F, Biancone L, Camussi G (2014). Urinary CD133+ extracellular vesicles are decreased in kidney transplanted patients with slow graft function and vascular damage. PLoS One.

[24] Zubiri I, Posada-Ayala M, Sanz-Maroto A, Calvo E, Martin-Lorenzo M, Gonzalez-Calero L (2014). Diabetic nephropathy induces changes in the proteome of human urinary exosomes as revealed by label-free comparative analysis. J Proteomics.

[25] Barutta F, Tricarico M, Corbelli A, Annaratone L, Pinach S, Grimaldi S (2013). Urinary Exosomal MicroRNAs in Incipient Diabetic Nephropathy. PLoS One.

[26] van Balkom BWM, Gremmels H, Ooms LSS, Toorop RJ, Dor FJMF, de Jong OG, et al. Proteins in preservation fluid as predictors of delayed graft function in kidneys from donors after circulatory death. Clin J Am Soc Nephrol. 2017;12:817–24. 10.2215/CJN.10701016 PMC5477220 28476951

[27] Gámez-Valero A, Monguió-Tortajada M, Carreras-Planella L, Franquesa M, Beyer K, Borràs FE (2016). Size-exclusion chromatography-based isolation minimally alters extracellular vesicles’ characteristics compared to precipitating agents. Sci Rep.

[28] de Menezes-Neto A, Sáez MJF, Lozano-Ramos I, Segui-Barber J, Martin-Jaular L, Ullate JME (2015). Size-exclusion chromatography as a stand-alone methodology identifies novel markers in mass spectrometry analyses of plasma-derived vesicles from healthy individuals. J Extracell Vesicles.

[29] Carreras-Planella L, Soler-Majoral J, Rubio-Esteve C, Lozano-Ramos SI, Franquesa M, Bonet J (2017). Characterization and proteomic profile of extracellular vesicles from peritoneal dialysis efflux. PLoS One.

[30] Miranda KC, Bond DT, Levin JZ, Adiconis X, Sivachenko A, Russ C (2014). Massively parallel sequencing of human urinary exosome/microvesicle RNA reveals a predominance of non-coding RNA. PLoS One.

[31] Fernandez-Llama P, Khositseth S, Gonzales PA, Star RA, Pisitkun T, Knepper MA (2010). Tamm-Horsfall protein and urinary exosome isolation. Kidney Int.

[32] Lozano-Ramos I, Bancu I, Oliveira-Tercero A, Pilar Armengol M, Menezes-Neto A, Del Portillo HA (2015). Size-exclusion chromatography-based enrichment of extracellular vesicles from urine samples. J Extracell Vesicles.

[33] Théry C, Clayton A, Amigorena S, Raposo G (2006). Isolation and characterization of exosomes from cell culture supernatants. Curr Protoc Cell Biol.

[34] Channavajjhalaa SK, Rossatoa M, Morandini F, Castagna A, Pizzolo F, Bazzoni F (2014). Optimizing the purification and analysis of miRNAs from urinary exosomes. Clin Chem Lab Med.

[35] Love MI, Huber W, Anders S (2014). Moderated estimation of fold change and dispersion for RNA-seq data with DESeq2. Genome Biol BioMed Central.

[36] Heberle H, Meirelles GV, da Silva FR, Telles GP, Minghim R (2015). InteractiVenn: a web-based tool for the analysis of sets through Venn diagrams. BMC Bioinformatics.

[37] Cox J, Mann M (2012). 1D and 2D annotation enrichment: a statistical method integrating quantitative proteomics with complementary high-throughput data. BMC Bioinformatics.

[38] Tyanova S, Temu T, Sinitcyn P, Carlson A, Hein MY, Geiger T (2016). The Perseus computational platform for comprehensive analysis of (prote)omics data. Nat Methods.

[39] Kim D-K, Kang B, Kim OY, Choi D, Lee J, Kim SR, et al. EVpedia: an integrated database of high-throughput data for systemic analyses of extracellular vesicles. J Extracell Vesicles. 2013;2 10.3402/jev.v2i0.20384. 10.3402/jev.v2i0.20384 PMC3760654 24009897

[40] Kim D-K, Lee J, Kim SR, Choi D-S, Yoon YJ, Kim JH (2015). EVpedia: a community web portal for extracellular vesicles research. Bioinformatics.

[41] Mathivanan S, Simpson RJ (2009). ExoCarta: a compendium of exosomal proteins and RNA. Proteomics.

[42] Keerthikumar S, Chisanga D, Ariyaratne D, Al Saffar H, Anand S, Zhao K (2015). ExoCarta: a web-based compendium of exosomal cargo. J Mol Bio.

[43] Kalra H, Simpson RJ, Ji H, Aikawa E, Altevogt P, Askenase P (2012). Vesiclepedia: a compendium for extracellular vesicles with continuous community annotation. PLoS Biol.

[44] Kozomara A, Griffiths-Jones S (2014). miRBase: annotating high confidence microRNAs using deep sequencing data. Nucleic Acids Res.

[45] Wong N, Wang X (2015). miRDB: an online resource for microRNA target prediction and functional annotations. Nucleic Acids Res.

[46] Remuzzi G, Cravedi P, Perna A, Dimitrov BD, Turturro M, Locatelli G (2006). Long-term outcome of renal transplantation from older donors. N Engl J Med.

[47] Trionfini P, Benigni A (2017). MicroRNAs as master regulators of glomerular function in health and disease. J Am Soc Nephrol.

[48] Forero A, So L, Savan R (2017). Re-evaluating strategies to define the Immunoregulatory roles of miRNAs. Trends Immunol.

[49] Antonieta Azancot M, Moreso F, Salcedo M, Cantarell C, Perello M, Torres IB (2014). The reproducibility and predictive value on outcome of renal biopsies from expanded criteria donors. Kidney Int.

[50] Piovesan AC, Lucon AM, David DSR, Nahas WC, Antonopoulos IM, Srougi M (2008). Multifocal Renal Allograft Biopsy: Impact on Therapeutic Decisions. Transplant Proc.

[51] Bouma HR, Ploeg RJ, Schuurs TA (2009). Signal transduction pathways involved in brain death-induced renal injury. Am J Transplant.

[52] Cheng L, Sun X, Scicluna BJ, Coleman BM, Hill AF (2014). Characterization and deep sequencing analysis of exosomal and non-exosomal miRNA in human urine. Kidney Int.

[53] Ohshima K, Inoue K, Fujiwara A, Hatakeyama K, Kanto K, Watanabe Y (2010). Let-7 MicroRNA Family Is Selectively Secreted into the Extracellular Environment via Exosomes in a Metastatic Gastric Cancer Cell Line. PLoS One.

[54] Huang X, Yuan T, Tschannen M, Sun Z, Jacob H, Du M (2013). Characterization of human plasma-derived exosomal RNAs by deep sequencing. BMC Genomics.

[55] van Balkom B, Eisele AS, Pegtel DM, Bervoets S, Verhaar MC (2015). Quantitative and qualitative analysis of small RNAs in human endothelial cells and exosomes provides insights into localized RNA processing, degradation and sorting. J Extracell Vesicles.

[56] Yu S, Huang H, Deng G, Xie Z, Ye Y, Guo R (2015). miR-326 targets antiapoptotic Bcl-xL and mediates apoptosis in human platelets. PLoS One.

[57] Li J, Li S, Chen Z, Wang J, Chen Y, Xu Z (2016). miR-326 reverses chemoresistance in human lung adenocarcinoma cells by targeting specificity protein 1. Tumour biol. Tumor Biol.

[58] Li Y, Gao Y, Xu Y, Ma H, Yang M (2015). Down-regulation of miR-326 is associated with poor prognosis and promotes growth and metastasis by targeting FSCN1 in gastric cancer. Growth Factors.

[59] Ji S, Zhang B, Kong Y, Ma F, Hua Y (2016). MiR-326 inhibits gastric cancer cell growth through down regulating NOB1. Oncol Res.

[60] Cao L, Wang J, Wang PQ (2016). MiR-326 is a diagnostic biomarker and regulates cell survival and apoptosis by targeting Bcl-2 in osteosarcoma. Biomed Pharmacother.

[61] Nemati E, Einollahi B, Lesan Pezeshki M, Porfarziani V, Reza Fattahi M, Pezeshki ML (2014). Does kidney transplantation with deceased or living donor affect graft survival?. Nephrourol Mon.

